# Prognostic Significance of Preoperative Neutrophil-to-Lymphocyte Ratio in Nonmetastatic Renal Cell Carcinoma: A Large, Multicenter Cohort Analysis

**DOI:** 10.1155/2016/5634148

**Published:** 2016-11-06

**Authors:** Seok-Soo Byun, Eu Chang Hwang, Seok Ho Kang, Sung-Hoo Hong, Jinsoo Chung, Tae Gyun Kwon, Hyeon Hoe Kim, Cheol Kwak, Yong-June Kim, Won Ki Lee

**Affiliations:** ^1^Department of Urology, College of Medicine, Seoul National University, Seoul, Republic of Korea; ^2^Department of Urology, College of Medicine, Chonnam National University, Gwangju, Republic of Korea; ^3^Department of Urology, College of Medicine, Korea University, Seoul, Republic of Korea; ^4^Department of Urology, College of Medicine, Catholic University, Seoul, Republic of Korea; ^5^Department of Urology, National Cancer Center, Goyang, Republic of Korea; ^6^Department of Urology, College of Medicine, Kyungpook National University, Daegu, Republic of Korea; ^7^Department of Urology, College of Medicine, Chungbuk National University, Cheongju, Republic of Korea; ^8^Department of Urology, College of Medicine, Hallym University, Chuncheon, Republic of Korea

## Abstract

*Background*. The prognostic significance of the neutrophil-to-lymphocyte ratio (NLR) in nonmetastatic renal cell carcinoma (non-mRCC) is controversial, although NLR has been established as a prognostic factor in several cancers. The objective of our study was to assess the prognostic significance of preoperative NLR in non-mRCC, based on a large, multicenter cohort analysis.* Methods*. Totally, 1,284 non-mRCC patients undergoing surgery were enrolled from six institutions between 2000 and 2014. Recurrence-free survival (RFS) and cancer-specific survival (CSS) were calculated, and the prognostic significance of NLR was evaluated.* Results*. Patients with higher NLR had larger tumors (*p* < 0.001), higher T stage (*p* < 0.001), worse Eastern Cooperative Oncology Group performance status (*p* < 0.001), worse symptoms (*p* = 0.003), sarcomatoid differentiation (*p* = 0.004), and tumor necrosis (*p* < 0.001). The 5-year RFS and CSS rates were significantly lower in patients with high NLR than in those with low NLR (each *p* < 0.001). Multivariate analysis identified NLR to be an independent predictor of RFS and CSS (each *p* < 0.05). Moreover, predictive accuracy of multivariate models for RFS and CSS increased by 2.2% and 4.2%, respectively, with NLR inclusion.* Conclusions*. Higher NLR was associated with worse clinical behavior of non-mRCC. Also, NLR was a significant prognostic factor of both RFS and CSS.

## 1. Introduction

Renal cell carcinoma (RCC) accounts for 3-4% of all adult malignancies, and its incidence rate has been steadily increasing worldwide [1]. In the United States, the estimated numbers of new cases and deaths in 2015 were 61,560 and 14,080, respectively [1]. Therefore, it is essential to optimize decision making in treatment and prognosis of RCC and to provide better counseling for each RCC patient. Until now, many characteristics of RCC itself and patients have been suggested as possible prognostic factors. However, only a few, including pathological stage and Fuhrman grade, are undisputed prognostic factors for RCC, especially nonmetastatic RCC (non-mRCC) [2].

Inflammation has an impact on tumorigenesis and tumor progression [3]. In addition, inflammation has been recently shown to predict the prognosis of various operable cancers [4]. As inflammation is easily accessible, can be measured reliably, and can be incorporated into the tumor staging system [4], its use as a prognostic factor seems promising.

Of the many hematological and biochemical markers for systemic inflammatory response, neutrophil-to-lymphocyte ratio (NLR) has been introduced relatively recently [5]. Neutrophils represent the inflammatory response, whereas lymphocytes reflect cell-mediated immunity [3]. Therefore, NLR may be a better indicator of inflammation compared to existing conventional markers. Furthermore, NLR is an inexpensive, easily accessible, and widely available marker. Initially, NLR was validated as a prognostic factor of major cardiac events [6, 7]. Since then, it has been established as a prognostic factor in several cancers including hepatocellular carcinoma and colorectal cancer [8–10].

Multiple studies suggested that NLR might be a prognostic factor in mRCC, irrespective of the treatment method [8, 11–13]. However, the few studies investigating the prognostic significance of NLR in non-mRCC have reported conflicting results [14–22]. Furthermore, previous studies were small-scale and lacked other possible prognostic factors as confounding variables (Table 1).

We assessed the prognostic significance of NLR in a large, multicenter cohort of non-mRCC patients. To our knowledge, this is the largest scale study conducted in the field, which also included the most widely accepted prognostic factors.

## 2. Patients and Methods

### 2.1. Patients

Approval for the study was obtained from the relevant institutional ethics committee. A total of 3,410 patients with RCC underwent curative partial or radical nephrectomy at six institutions between 2000 and 2014. We consecutively excluded 239 patients with lymph node and/or distant metastasis immediately after surgery, 574 patients who did not have any of the three major RCC subtypes (clear cell, papillary, and chromophobe variants), 351 patients with postoperative follow-up durations within 3 months, and 962 patients with unavailable data on at least one of the relevant parameters. Only patients with complete absolute neutrophil count (ANC) and absolute lymphocyte count (ALC) data within the 2 weeks before surgery were included in the study. Finally, 1,284 non-mRCC patients (pathologically, TxN0M0) from any of the three major RCC subtypes were included in this study and retrospectively reviewed.

### 2.2. Variables

The characteristics of RCC and patients are detailed in Table 2.

For most patients, postoperative follow-up was scheduled every 3 months for 6 months, every 6 months for the next 3 years, and yearly thereafter. NLR was defined as the ANC divided by the ALC. The general health status was determined by the Eastern Cooperative Oncology Group performance status (ECOG PS). Tumor size was measured as the greatest diameter of the pathologic specimen. Pathologic staging was performed using the 2002 tumor-node-metastasis (TNM) classification system, and grading was performed using Fuhrman nuclear grading system. The histologic subtype was determined using the 2004 World Health Organization (WHO) international histological classification of tumors. For all specimens, urologic pathologists of each institution determined the pathologic features of the tumor. Recurrence-free survival (RFS) and cancer-specific survival (CSS) were calculated from the date of surgery to the date of recurrence and RCC-specific death, respectively, and were confirmed by imaging studies.

### 2.3. Statistical Analysis

The primary endpoints were RFS and CSS. The ideal cut-off level of NLR was estimated by testing all possible cut-off levels that were likely to discriminate between survival and recurrence and RCC-specific death, using the Cox proportional hazard model. The ideal cut-off level determined was then rounded to clinically relevant levels [11]. To compare the relationship between the characteristics of RCC and the patients, Student* t-*test, Pearson chi-squared test, or Fisher exact test stratified by NLR was used.

The RFS and CSS rates were calculated using the Kaplan-Meier method stratified by NLR, and the log-rank test was used to compare the groups. The prognostic significance of NLR as a continuous and categorical variable was evaluated using variables entered into the Cox proportional hazards model. The variables analyzed included patient age, gender, body mass index (BMI), ECOG PS, symptoms at presentation, tumor size, pathologic T stage, Fuhrman grade, histologic subtype, sarcomatoid differentiation, and tumor necrosis. The accuracy of NLR in predicting RFS and CSS was reflected by Harrell concordance index (c-index) calculated using the Cox proportional hazard models with and without the incorporation of NLR.

All tests were two-sided, and *p* < 0.05 was considered statistically significant. Survival, the Cox regression method in R 3.2.2 (R Development Core Team, Vienna, Austria, https://www.R-project.org/) was used to calculate the c-index, whereas IBM SPSS Statistics for Windows, version 21.0 (IBM Corp., Armonk, NY, USA) was used for other statistical assessments.

## 3. Results

### 3.1. The Association between Clinical and Pathologic Characteristics and NLR

A cut-off NLR level of 3.7 was estimated to be the optimal cut-off level for discriminating between patients' recurrences (hazard ratio (HR) = 3.049, 95% confidence interval (CI) = 2.015–4.614, and *p* < 0.001). The same NLR cut-off level was effective for discriminating between patients' RCC-specific deaths (HR = 4.947, 95% CI = 2.766–8.849, and *p* < 0.001). Based on these results, the NLR cut-off level of 3.7 was used in all subsequent analyses (low NLR, <3.7; high NLR, ≥3.7).

The mean follow-up period was 46.8 months for all patients (median 39 months; interquartile range, 19–69 months). The mean NLRs of patients with low and high NLR were 1.8 ± 0.7 and 6.0 ± 3.2, respectively (*p* < 0.001). Table 1 shows the association of NLR with different clinical and pathological characteristics. Patients with high NLR differed significantly from those with low NLR in various parameters. Patients with high NLR were older (*p* = 0.001) and had higher ECOG PS (*p* < 0.001) and T stage (*p* < 0.001) and larger tumors (*p* < 0.001) compared to those with low NLR. Patients with high NLR also had greater symptom ratios (*p* = 0.003), sarcomatoid differentiation ratios (*p* = 0.004), and tumor necrosis ratios (*p* < 0.001).

### 3.2. Recurrence-Free Survival in relation to NLR

**Figure 1:**
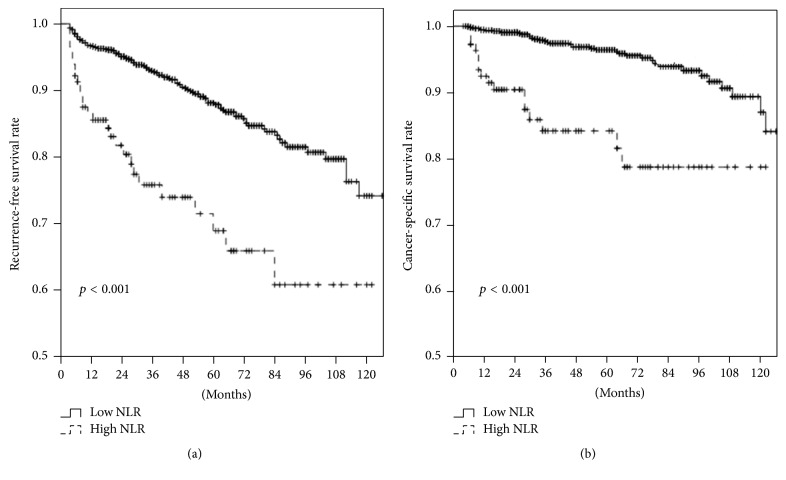
Kaplan-Meier curve for recurrence-free survival (a) and cancer-specific survival (b) for patients with nonmetastatic renal cell carcinoma according to neutrophil-to-lymphocyte ratio. NLR, neutrophil-to-lymphocyte ratio.

During follow-up, 142 (11.1%) patients had recurrence (Table 2). The 5-year RFS rates were 71.6% in patients with high NLR and 88.2% in those with low NLR. The 5-year RFS rate was significantly lower in patients with high NLR than in those with low NLR (*p* < 0.001; Figure 1(a)).

Multivariate analysis identified NLR to be an independent predictor of RFS (HR of NLR as a continuous variable = 1.081, *p* = 0.028; HR of NLR as a categorical variable = 1.788, *p* = 0.009; Table 3). The predictive accuracy of the multivariate model with NLR was 81.1%, whereas that of the multivariate model without NLR was 78.9%.

### 3.3. Cancer-Specific Survival in relation to NLR

During follow-up, 56 (4.4%) patients died of RCC-related causes (Table 2). The 5-year CSS rates were 84.2% in patients with high NLR and 96.4% in those with low NLR. The 5-year CSS rate was significantly lower in patients with high NLR than in those with low NLR (*p* < 0.001; Figure 1(b)).

Multivariate analysis identified NLR to be an independent predictor of CSS (HR of NLR as a continuous variable = 1.156, *p* = 0.009; HR of NLR as a categorical variable = 2.566, *p* = 0.004; Table 4). The predictive accuracy of the multivariate model with NLR was 87.9%, whereas that of the multivariate model without NLR was 83.7%.

## 4. Discussion

In this study, NLR was identified to be a significant prognostic factor of both RFS and CSS in patients with non-mRCC, even when the models were adjusted for other well-known prognostic factors. The predictive accuracy of the multivariate models for RFS and CSS increased by 2.2% and 4.2%, respectively, with NLR inclusion.

The present study had several strengths, compared to the previous studies in the field (Table 1). Firstly, this was the largest study that included the three major histologic subtypes of RCC. Secondly, while the present study evaluated both RFS and CSS, most of the previous studies did not evaluate CSS. The identification of CSS as well as RFS is a corner stone to prove the prognostic value of NLR. Finally, the present study included the most widely accepted independent prognostic factors of non-mRCC, including age, gender, and BMI; ECOG PS; symptoms at presentation; tumor size, stage, and grade; histologic subtype, sarcomatoid differentiation, and tumor necrosis.

In terms of clinical and pathologic characteristics at diagnosis, patients with high NLR differed significantly from those with low NLR in various parameters. Patients with high NLR had a larger tumor, a higher T stage, worse ECOG PS, worse symptoms, sarcomatoid differentiation, and tumor necrosis. These results are similar to those reported in previous studies [17, 18, 20], suggesting that higher NLR may be associated with worse clinical behavior of non-mRCC.

NLR was shown to be a possible prognostic factor for mRCC in multiple studies, irrespective of the treatment method [8, 11–13]. However, studies concerning the prognostic significance of NLR for non-mRCC are scarce, with conflicting results. Some studies did not show a relationship between NLR and non-mRCC prognosis [16, 22], while others did [14, 15, 18–21]. Interestingly, one study reported different results for RFS and CSS [17]. These conflicting results may partly be because previous studies were relatively small-scale and lacked other possible prognostic factors as confounding variables (Table 1).

An important point is that most of the previous studies incorporated NLR as a categorical variable in their models. The use of a continuous variable reflects an intrinsic effect, whereas that of a categorical variable seems to adjust itself and to be created [23]. In addition, it is difficult to interpret the prognostic value of NLR using different cut-off levels, although most studies including the present one showed that the cut-off levels of NLR were in the range 3-4 (Table 1). In this respect, it is remarkable that NLR was not only used as a categorical variable but also as a continuous variable in this study. We identified that NLR as a continuous variable was also an independent prognostic factor. Interestingly, NLR cut-off level of 3.7 was estimated for CSS as well as RFS in this study. Considering that CSS is in alignment with RFS in non-mRCC, these results may strengthen our conclusion.

It is well known that inflammation affects tumorigenesis and progression [3, 17]. Neutrophils represent the inflammatory response, whereas lymphocytes reflect cell-mediated immunity [3]. Therefore, a high NLR reflects both an increased inflammatory and a decreased antitumor immune response, suggesting a possible contribution to aggressive tumor biology, tumor progression, and poor survival [17]. In various cancers including hepatocellular carcinoma and colorectal cancer, high NLR was associated with poor outcome [9, 10]. This was also supported by the results of our clinical study, which showed that higher NLR was likely to be associated with worse clinical behavior and indicated poor prognosis for RFS and CSS.

In contrast to our findings, some studies did not show a relationship between NLR and non-mRCC prognosis [16, 22]. In a study of 678 patients with cRCC, Pichler et al. [16] reported that NLR was not an independent prognostic factor for CSS or metastasis-free survival. However, NLR was only included as a categorical variable in this analysis. Certainly, a specified cut-off level may create a false or misleading association. Furthermore, they only analyzed patients with cRCC. As RCC is a heterogeneous and complex disease [24, 25], its results may not be directly applicable to patients with non-cRCC. In a study of 228 patients with non-mRCC, Jagdev et al. [22] reported that NLR was not an independent prognostic factor for disease-free survival. However, their study involved only a small number of patients. Furthermore, as their study did not focus on NLR, the data on NLR were insufficient and were logarithmically transformed for analysis.

This study also had a few limitations. Firstly, data were retrospectively collected. Secondly, preoperative conditions such as chronic infection and chronic disease, which might affect the level of NLR, were not included. However, it is impossible to identify all the conditions associated with the NLR level in the clinical setting. Therefore, this study may be a better representation of the prognostic significance of NLR in actual practice. Lastly, this study lacked a central review of pathology, although most of the previous large multicenter studies did. Instead, urologic pathologists determined all pathologic features at each institution.

Despite limitations, it is noted that this study is the largest in the field, incorporating the most widely accepted independent prognostic factors of non-mRCC and evaluating both RFS and CSS.

## 5. Conclusion

This study showed that patients with high NLR differed significantly from those with low NLR in various clinical and pathologic parameters, suggesting that higher NLR may indicate worse clinical behavior of non-mRCC. In addition, NLR was a significant prognostic factor of both RFS and CSS, and incorporation of NLR into conventional prognostic predictors increased the predictive accuracy by 2.2% and 4.2%, respectively. This study suggests that the use of preoperative NLR may be helpful in counseling and clinical trial design in patients with non-mRCC.

## Figures and Tables

**Table 1:** Main characteristics of recently published studies on prognostic value of neutrophil-to-lymphocyte ratio in patients with nonmetastatic renal cell carcinoma.

Study cohort	Study cases	Histologic subtype	TNM stage	NLR				
				Value	Cut-off	Prognostic significance^*∗*^		Adjustment variables
						RFS^#^	CSS	
Lucca et al. [15]	430	Clear cell	T1–3	Median 2.9	4.2	Yes	NA	Stage, grade, tumor size, necrosis
Pichler et al. [16]	678	Clear cell	T1–4	Mean 3.51	3.3	No	No	Age, gender, stage, grade, necrosis
Viers et al. [17]	827	Clear cell	M0	Median 3.51	4.0	No	Yes	Age, gender, ECOG PS, tumor size, Sx, stage, grade, necrosis
Huang et al. [18]	218	Papillary	T1–3Nx	Median 3.1	3.6	Yes	NA	Age, gender, Sx, DM, HTN, stage, node, TNM group, grade, necrosis, ANC, ALC
De Martino et al. [19]	281	Papillary and chromophobe	T1–3Nx	Median 2.6	3.6	Yes	NA	Age, gender, ECOG PS, stage, TNM group, grade, MVI, ANC, ALC
Wen et al. [20]	327	All	T1–4	Mean 2.72	1.7	Yes	NA	Age, gender, tumor size, stage, subtype
Forget et al. [21]	227	All	M0	Median 3.01	5.0	Yes	NA	Age, gender, stage, grade, node
Jagdev et al. [22]	228	3 major subtypes	M0	NA	NA	No	NA	NA
Present study	1,284	3 major subtypes	T1–4	Mean 2.2	3.7	Yes	Yes	Age, gender, BMI, ECOG PS, Sx, tumor size, stage, grade, subtype, sarcomatoid differentiation, necrosis

^*∗*^Results from multivariate analysis.

^#^RFS stands for disease-free, progression-free, and metastasis-free survival as well as recurrence-free survival.

TNM, tumor-node-metastasis; NLR, neutrophil-to-lymphocyte ratio; RFS, recurrence-free survival; CSS, cancer-specific survival; necrosis, tumor necrosis; NA, not available; ECOG PS, Eastern Cooperative Oncology Group performance status; MVI, microvascular invasion; ANC, absolute neutrophil count; ALC, absolute lymphocyte count; Sx, symptoms at presentation; DM, diabetes mellitus; HTN, hypertension.

**Table 2:** Association of different clinical and pathological characteristics with neutrophil-to-lymphocyte ratio in patients with nonmetastatic renal cell carcinoma.

Variable	All	Low NLR	High NLR	*p* value
Number of subjects	1,284	1,168	116	
NLR, mean ± SD	2.2 ± 1.7	1.8 ± 0.7	6.0 ± 3.2	<0.001^*∗*^
Age, mean ± SD, year	55.9 ± 12.9	55.5 ± 12.8	59.8 ± 12.9	0.001^*∗*^
Gender				0.236^*∗∗*^
Male, *n* (%)	913 (71.1)	825 (70.6)	88 (75.9)	
Female,* n* (%)	371 (28.9)	343 (29.4)	28 (24.1)	
BMI, mean ± SD, kg/m^2^	24.6 ± 3.3	24.7 ± 3.2	23.8 ± 3.4	0.006^*∗*^
ECOG PS ≥ 1,* n* (%)	180 (14.0)	148 (12.7)	32 (27.6)	<0.001^*∗∗*^
Symptoms at presentation				0.003^*∗∗*^
No symptom,* n* (%)	975 (75.9)	900 (77.1)	75 (64.7)	
Symptom,* n* (%)	309 (24.1)	268 (22.9)	41 (35.3)	
Tumor size				
(1) mean ± SD, cm	4.08 ± 2.68	3.94 ± 2.54	5.50 ± 3.55	<0.001^*∗*^
(2) Category				<0.001^*∗∗*^
<4 cm,* n* (%)	748 (58.3)	701 (60.0)	47 (40.5)	
4–7 cm,* n* (%)	351 (27.3)	321 (27.5)	30 (25.9)	
≥7 cm,* n* (%)	185 (14.4)	146 (12.5)	39 (33.6)	
Side				1.000^*∗∗∗*^
Unilateral,* n* (%)	1,268 (98.8)	1,153 (98.7)	115 (99.1)	
Bilateral,* n* (%)	16 (1.2)	15 (1.3)	1 (0.9)	
Type of nephrectomy				<0.001^*∗∗*^
Radical,* n* (%)	634 (49.4)	552 (47.3)	82 (70.7)	
Partial,* n* (%)	650 (50.6)	616 (52.7)	34 (29.3)	
Method of surgery				0.042^*∗∗*^
Open,* n* (%)	697 (54.3)	628 (53.8)	69 (59.5)	
Laparoscopic,* n* (%)	316 (24.6)	283 (24.2)	33 (28.4)	
Robot,* n* (%)	271 (21.1)	257 (22.0)	14 (12.1)	
T stage				<0.001^*∗∗*^
T1,* n* (%)	1,016 (79.1)	945 (80.9)	71 (61.2)	
T2,* n* (%)	89 (6.9)	75 (6.4)	14 (12.1)	
T3-4,* n* (%)	179 (13.9)	148 (12.7)	31 (26.7)	
Fuhrman's grade				0.561^*∗∗*^
G1-2,* n* (%)	664 (51.7)	607 (52.0)	57 (49.1)	
G3-4,* n* (%)	620 (48.3)	561 (48.0)	59 (50.9)	
Histologic subtype				0.042^*∗∗*^
Clear cell,* n* (%)	1,114 (86.8)	1,017 (87.1)	97 (83.6)	
Papillary,* n* (%)	87 (6.8)	73 (6.3)	14 (12.1)	
Chromophobe,* n* (%)	83 (6.5)	78 (6.7)	5 (4.3)	
Sarcomatoid differentiation, yes,* n* (%)	29 (2.3)	22 (1.9)	7 (6.0)	0.004^*∗∗*^
Tumor necrosis, yes,* n* (%)	208 (16.2)	174 (14.9)	34 (29.3)	<0.001^*∗∗*^
Recurrence,* n* (%)	142 (11.1)	114 (9.8)	28 (24.1)	<0.001^*∗∗*^
RCC-specific death,* n* (%)	56 (4.4)	40 (3.4)	16 (13.8)	<0.001^*∗∗*^

NLR, neutrophil-to-lymphocyte ratio; low NLR, <3.7; high NLR, ≥3.7; BMI, body mass index; ECOG PS, Eastern Cooperative Oncology Group performance status; RCC, renal cell carcinoma; *n*, number of subjects; SD, standard deviation.

^*∗*^Student *t*-test.

^*∗∗*^Pearson's chi-square test.

^*∗∗∗*^Fisher's exact test.

**Table 3:** Multivariate analyses predicting probability of cancer recurrence in relation to the neutrophil-to-lymphocyte ratio in patients with nonmetastatic renal cell carcinoma.

Variables	NLR as a continuous variable			NLR as a categorical variable		
	HR	95% CI	*p* value	HR	95% CI	*p* value
Age	1.011	0.997–1.025	0.134	1.011	0.997–1.026	0.123
Gender						
Female versus male	0.873	0.588–1.296	0.502	0.876	0.591–1.299	0.510
BMI	0.959	0.907–1.015	0.146	0.959	0.907–1.014	0.146
ECOG PS						
≥1 versus 0	1.936	1.270–2.950	0.002	1.900	1.244–2.902	0.003
Symptoms at presentation	1.185	0.811–1.731	0.380	1.208	0.830–1.758	0.325
Tumor size	1.011	1.005–1.017	0.001	1.011	1.004–1.017	0.001
T stage			0.009			0.010
T2 versus T1	1.384	0.745–2.571	0.303	1.376	0.743–2.550	0.310
T3-4 versus T1	2.068	1.281–3.340	0.003	2.050	1.267–3.314	0.003
Fuhrman's grade						
G3-4 versus G1-2	1.974	1.352–2.882	<0.001	1.958	1.340–2.863	0.001
Histologic subtype			0.012			0.019
pRCC versus cRCC	1.044	0.582–1.872	0.886	1.029	0.575–1.841	0.924
chRCC versus cRCC	0.104	0.023–0.467	0.003	0.132	0.032–0.545	0.005
Sarcomatoid differentiation	2.095	1.061–4.137	0.033	2.004	1.010–3.977	0.047
Tumor necrosis	1.255	0.817–1.927	0.300	1.265	0.825–1.939	0.282
NLR						
(1) Continuous	1.081	1.009–1.160	0.028			
(2) High versus low NLR				1.788	1.153–2.771	0.009

NLR, neutrophil-to-lymphocyte ratio; low NLR, <3.7; high NLR, ≥3.7; BMI, body mass index; ECOG PS, Eastern Cooperative Oncology Group performance status; cRCC, clear cell renal cell carcinoma; pRCC, papillary renal cell carcinoma; chRCC, chromophobe renal cell carcinoma; HR, hazard ratio; CI, confidence interval.

**Table 4:** Multivariate analyses predicting probability of cancer-specific death in relation to the neutrophil-to-lymphocyte ratio in patients with nonmetastatic renal cell carcinoma.

Variables	NLR as a continuous variable			NLR as a categorical variable		
	HR	95% CI	*p* value	HR	95% CI	*p* value
Age	1.042	1.016–1.069	0.002	1.044	1.018–1.072	0.001
Gender						
Female versus male	0.652	0.324–1.313	0.231	0.648	0.323–1.300	0.222
BMI	0.916	0.832–1.009	0.074	0.924	0.840–1.017	0.105
ECOG PS						
≥1 versus 0	2.820	1.498–5.309	0.001	2.672	1.408–5.071	0.003
Symptoms at presentation	1.029	0.558–1.897	0.927	1.056	0.577–1.932	0.860
Tumor size	1.012	1.002–1.022	0.015	1.012	1.002–1.022	0.018
T stage			0.022			0.020
T2 versus T1	0.665	0.198–2.233	0.509	0.662	0.198–2.215	0.503
T3-4 versus T1	2.175	1.025–4.617	0.043	2.209	1.041–4.688	0.039
Fuhrman's grade						
G3-4 versus G1-2	2.155	1.141–4.072	0.018	2.101	1.110–3.977	0.023
Histologic subtype			0.854			0.860
pRCC versus cRCC	1.268	0.551–2.919	0.576	1.257	0.554–2.850	0.584
chRCC versus cRCC	0.001	<0.001–5.496	0.959	0.001	<0.001–6.687	0.962
Sarcomatoid differentiation	3.355	1.230–9.148	0.018	3.092	1.123–8.514	0.029
Tumor necrosis	1.054	0.509–2.181	0.888	1.097	0.537–2.242	0.799
NLR						
(1) Continuous	1.156	1.037–1.289	0.009			
(2) High versus low NLR				2.566	1.348–4.887	0.004

NLR, neutrophil-to-lymphocyte ratio; low NLR, <3.7; high NLR, ≥3.7; BMI, body mass index; ECOG PS, Eastern Cooperative Oncology Group performance status; cRCC, clear cell renal cell carcinoma; pRCC, papillary renal cell carcinoma; chRCC, chromophobe renal cell carcinoma; HR, hazard ratio; CI, confidence interval.

## Abbreviations

Non-mRCC: Nonmetastatic renal cell carcinoma
NLR: Neutrophil-to-lymphocyte ratio
ANC: Absolute neutrophil count
ALC: Absolute lymphocyte count
ECOG PS: Eastern Cooperative Oncology Group performance status
TNM: Tumor-node-metastasis
WHO: World Health Organization
RFS: Recurrence-free survival
CSS: Cancer-specific survival
BMI: Body mass index
HR: Hazard ratio
CI: Confidence interval.
